# OpenSEA: a 3D printed planetary gear series elastic actuator for a compliant elbow joint exoskeleton

**DOI:** 10.3389/frobt.2025.1528266

**Published:** 2025-02-28

**Authors:** Benjamin Jenks, Hailey Levan, Filip Stefanovic

**Affiliations:** ^1^ Department of Biomedical Engineering, University at Buffalo, Buffalo, NY, United States; ^2^ Department of Mechanical Engineering, University at Buffalo, Buffalo, NY, United States; ^3^ Biomedical and Mechanical Engineering, Messiah University, Mechanicsburg, PA, United States

**Keywords:** series elastic actuator, 3D printed exoskeleton, rehabilitation, compliant joint, assistive robotics

## Abstract

**Introduction:**

Next-generation assistive robotics rely on series elastic actuators (SEA) that enable compliant human-robot interaction. However, currently there is a deficiency of openly available SEA systems to support this development. To address this, we propose a novel design of a compliant 3D-printed SEA device for elbow movement rehabilitation exoskeletons that we make openly available.

**Methods:**

We designed a 3D-printed SEA to incorporate a planetary gear system and torsional spring, offering compliance, adaptability, and cost-effectiveness. The design provides a high-power density, that can address torque limitations in 3D printed SEA systems. Our design utilizes a 4.12 Nm motor operating at 26 RPM based on assessment of functional performance differences across healthy and post-stroke individuals. Moreover, the design of this SEA allows for easily adjustable parameters to fit different joints, or various torque output configurations, in low-cost exoskeleton applications in rehabilitation.

**Results:**

Testing demonstrated an average compliance contribution of the planetary gear and the average total system compliance of 14.80° and 22.22°, respectively. This range conforms to those expected in human-exoskeleton interaction. Similarly, an FEA analysis of the 3D printed system shows stress ranges of the SEA gears to be between 50 and 60.2 MPa, which causes a displacement of approximately 0.14 mm. This is within the operational flexural range of standard 3D printed materials such as PLA, which is 175 MPa.

**Discussion:**

The study demonstrates an openly available SEA design for 3D printed exoskeletons. This work provides an entry point for accessible exoskeleton design, specifically for rehabilitation. Future work will explore the role of segment vs joint rigidity in developing next-generation compliant exoskeletons, and improving accessibility for personalizable assistive exoskeletons. All designs presented herein are publicly available.

## 1 Introduction

Exoskeletons are expected to have a transformative impact on human motor control rehabilitation applications. While exoskeletons are not yet widely used in rehabilitation, their proven benefits have generated increasing patient interest in finding facilities that employ this technology (Hohl et al., 2022). It is expected that the rehabilitation exoskeleton market will grow to $4 billion by 2028, at a compound annual growth rate of 38%, showing significant growth in its application to support future neuromuscular healthcare practices. A major barrier to the implementation of this technology is the cost and personalization of exoskeletons which continue to impact usage and accessibility–a single exoskeleton can cost between $50,000 - $150,000 (Pinto et al., 2023). Due to this limitation, 3D printed exoskeletons can be designed to create more customizable and low-cost options to support rehabilitation in less economically developed areas. However, the majority of these 3D printed exoskeletons are rigid structures that lack the ability to conform to the complex dynamics and compliance of human joints (Chirila et al., 2020; Batkuldinova et al., 2021; Esposito et al., 2022; Rojek et al., 2023; Dudley et al., 2021). Similarly, exoskeletons can be notoriously difficult to design due to significant variability across subjects, complex approaches to comfort and attachment, and a lack of personalization (Bengler et al., 2023; Siviy et al., 2023; Herr, 2009). To address this gap, we present a 3D printable compliant joint that we apply as part of an elbow exoskeleton, and demonstrate the characteristics of its use in motor rehabilitation. By developing the OpenSEA, we aim to provide a template for openly available mechanical designs that support customizable compliant actuated joints for improved human-robot interaction in assistive robotics.

For their effective use, upper limb exoskeletons must provide postural support to the user, along with scalable performance characteristics. However, overly rigid exoskeletons can impede recovery by increasing difficulty, metabolic cost, and modified movement dynamics or range of movement in outcomes (Nuckols and Sawicki, 2020; Moeller et al., 2022). Recently, it has been shown that ‘soft’ exoskeletons can improve functional support and usability, since they provide a more natural interface between the robotic device and the human body. These so called, “soft” exoskeletons exist in various forms such as rigid structures with compliant actuators, soft structures with rigid actuators, or soft structures with compliant actuators (Sanchez-Villamañan et al., 2019).

Compliant exoskeletons are essential for compatibility with human biomechanics and dynamics (Vanderborght et al., 2013; Park et al., 2023; Gálvez-Zúñiga and Aceves-López, 2016). For example, when supporting elbow flexion and extension rehabilitation with exoskeletons, there are several challenges. The elbow joint is among the most complex joints in the body, as it is a synovial hinge with three articulations (i.e., ulnohumeral, radiohumeral, and proximal radioulnar) along with numerous muscles that attach or cross the elbow joint (Card and Lowe, 2024). Moreover, effective rehabilitation requires functional repetition of elbow movements, which can exacerbate fatigue or strain in the joint (Kawahira et al., 2010; Yoon and Shin, 2024). In this way, tailoring interventions to an individual’s biomechanical needs is crucial for maximizing effectiveness. Thus, by designing compliant exoskeletons that are consistent with the complex movements of the joint, secondary negative effects can be reduced, and ultimately provide more user-friendly and effective recovery modalities.

Recently, several compliant exoskeletons have been developed to address some of these issues. For example, series elastic actuators (SEA) have been designed to provide compliant actuation of joints when interfacing robotic systems with the human body. The principle of SEAs relies on the use of series elasticity, which follows Hooke’s Law, to absorb forces and release stored energy back to the output. This ensures a linear behavior of elastic materials, enabling precise force control relative to the position of the actuator. While novel elastic materials, air compression or magnetic forces can be used to achieve this behavior, mechanical springs remain the most common choice for implementation.

Lee and Oh used a harmonic drive coupled with a torsional spring to create a more dynamic response in ground force reactions for a robotic leg (Lee and Oh, 2019). They demonstrated that they could more effectively control the actuator torque in tracking performance while controlling the limb. Similar results are reported in other more recent studies as well (Marconi et al., 2019; Sarkisian et al., 2023; Kang et al., 2022). Thus, SEAs provide an effective compliant mechanism that allows for robust torque control in human-machine interaction. These are becoming ever more common in applications such exoskeletons as well as prosthetic devices.

Similarly, Chen and colleagues developed a SEA and cable-driver differential (Chen et al., 2019) that provided high resolution torque control and impedance in a low-weight framework. Their design demonstrated a sigmoidal/exponential stiffness profile, allowing for a dynamic stiffness in the actuator based on the size of joint deflection. This is an important behavior in the SEA, since it allows for robust joint stiffness depending on the characteristics of the movement. Additionally, rotary SEAs have shown similar characteristics in non-linear stiffness profiles, providing robust applications for use in exoskeletons and joint control (Zhou et al., 2021; Kong et al., 2010).

Despite their promising results and broad utility in robotic actuation, there are some problems with the use of SEAs in complex joint control. For example, SEAs can suffer from torque (or other performance) limitations due to the elastic spring, which reduces the torque output of the system (Lee and Oh, 2022). Thus, the torque bandwidth of the SEA system can be limited depending on the velocity, gear ratio, or damping effect of the mechanical system. Despite this drawback, there are methods that can minimize the damping of the motor (e.g., apply high controller gain or reducing stiffness profile, etc.). What is more, is that these SEAs are often difficult to manufacture, and should be personalized to user’s unique physiology.

We hypothesize that a new way to address some of these limitations is by creating a novel 3D printed planetary gear-based SEA system. Specifically, planetary gears are known for providing a high-power density (e.g., high torque output in compact form factor), which we believe can address the torque limitations of other SEAs. In this paper, we develop the 3D printed planetary gear SEA for elbow movement rehabilitation. The inherent elasticity of the actuator design can provide high-torque transfer in a compact design and allow for personalization of the transmission effect based on the gear characteristics. We develop this system as an openly available 3D printed series elastic actuator, to provide a low-cost, high-power density solution for modern applications in functional rehabilitation.

This paper is organized such that the mechanical design theory and methods are presented first, along with fabrication details–all design files are linked to our GitHub repository. We then describe how we test the device, and provide the results of performance test characteristics of the SEA compliant joint. We demonstrate that a 3D printed SEA joint can be developed for use in low-cost exoskeleton applications, especially in upper limb applications in rehabilitation.

## 2 Materials and methods

The section is organized as follows - the biomechanical requirements of an exoskeleton supported movement are calculated such that torque, loading, and other dynamic details are defined. Following this, the SEA principles and exoskeleton mechanical designs are described (e.g., gears, motors, elastic elements, etc.), along with the fabrication, assembly, and operational conceptualization. Based on the intended operation, we provide an overview of static and compliant performance testing for the SEA/exoskeleton which include a description of why the tests are important, as well as how the tests will be completed.

### 2.1 Mechanical design

#### 2.1.1 Torque assessment

Since our exoskeleton is designed to assist with elbow rehabilitation, we design the torque requirements so that the generated joint activity resembles that of natural human motion. To do this, we use torques at the elbow from healthy and post-stroke individuals based on previously reported data. By estimating the difference in torque between these two states, we define an operational range for our system. However, since many post-stroke patients have a large variability in body segment usability, weight, mass, and length, the amount of torque applied at the elbow can also vary (Van Dokkum et al., 2014) and so we need to take into account this variability.

#### 2.1.2 Human properties

We estimate elbow torque in healthy and post-stroke individuals using average body segments lengths (Table 1) as measured by Harless, and reported by Drillis (Drillis et al., 1964). Patients with impaired motor function often experience a decrease in both movement speed and joint strength (torque) of the affected limbs. Research suggests an average torque reduction after a stroke to be 63% in elbow extension (Lum et al., 2004). This is used to approximate the torque assistance needed by our elbow exoskeleton. By incorporating these torque loss values into the calculations, a more realistic model of the elbow joint function can be created.

**TABLE 1:** Reported average body segment measurements.

	Average body segment values	
	Length (cm)	Weight (g)
Hand	20.3	540
Forearm	29.9	1,160
Upper Arm	36.3	2070

The patient’s center of mass (COM) also plays a vital role in determining the torque generated at the elbow. The distance between the COM and the elbow joint influences the effect of gravity on the forearm and hand. The data in Table 2 – as reported in Adolphe et al. (2017) – provide the average COM values for males and females, which were used for further calculations.

**TABLE 2:** Percentage of center of mass.

	COM (%)		
	Male	Female	Average
Hand	79.00	74.74	76.87
Forearm	45.74	45.59	45.67
Upper Arm	57.72	57.54	57.63

Muscles exert force on bones through their insertion points, which are the locations where the muscles and tendons attach. These insertion points define the spatial location of the forces acting on the forearm during the flexion and extension movements. In addition, a typical moment arm value of 2.5 cm, as reported in Moritomo et al. (2007), was used to represent the average muscle insertion points across different arm positions.

#### 2.1.3 Torque calculations

**FIGURE 1:**
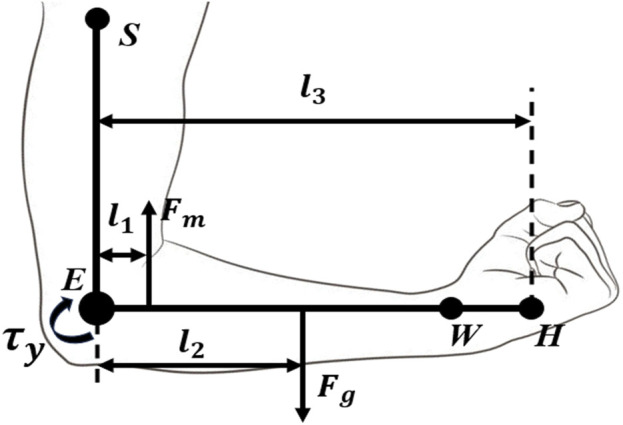
Free-body diagram used to calculate the torque about the elbow, showing the shoulder (S), elbow (E), wrist (W), and hand (H).

To evaluate the forces acting on the body in this scenario, a free-body diagram was used to represent the forces affecting the forearm during elbow flexion and extension (Figure 1). The key lengths and forces are: $l_{1}$ : Length of the muscle insertion point; $l_{2}$ : Distance of the center of mass from the elbow joint; $l_{3}$ : The total length of the forearm; $F_{m}$ : The force of the flexion/extension exerted by muscles; $F_{g}$ : Force of gravity; $\tau_{y}$ : Uniaxial torque about the elbow.

The center of mass (COM) of the forearm can be calculated using Equation 1, where $m_{1}$ and $m_{2}$ represent the mass of the forearm and hand, respectively. Then $x_{1}$ and $x_{2}$ represent the distances of the forearm and hand COMs from the elbow joint, respectively.

$$COM=\frac{m_{1}x_{1}+m_{2}x_{2}}{m_{1}+m_{2}}$$ (1)

Anatomical proportionality was used to determine lengths *x*_1_ and *x*_2_, from a previous study (Adolphe et al., 2017). It is known that 45.665% and 76.87% are the percentages of the total length of the arm to the COM for the forearm and hand from the elbow joint, respectively. Using these percentages and the average forearm length data from another study (Drillis et al., 1964) (adjusted for sex), the COM was calculated for the entire forearm. Using Figure 1, we have the following relationships:

$$\sum \tau_{y}=F_{m}l_{1}-F_{g}l_{2}=0$$ (2)

$$F_{m}=\frac{F_{g}l_{2}}{l_{1}}$$ (3)

$$\tau_{y}=F_{m}l_{1}$$ (4)

From Equation 3, the force exerted by the muscles can be determined, and this is substituted into Equation 4 to obtain the total torque produced about the elbow. We also add a mass of 35 g to account for the mass of the exoskeleton that incurs an added torque effect. To determine the amount of torque required to assist the user, the percentages reported in Lum et al. (2004) were included.

$$\tau_{lost}={0.63\tau }_{y}$$ (5)

Using Equations 1–5 the following results in Table 3 were obtained.

**TABLE 3:** Results and values for the length, COM, force, and torque.

Variable	Value
$l_{1}$	2.5 cm
$l_{2}$	27.89 cm
$l_{3}$	50.2 cm
$x_{1}$	22.9 cm
$x_{2}$	38.6 cm
$F_{m}$	189.88 N

#### 2.1.4 Electric actuator

To meet our torque requirements, we selected a 12V, 26 RPM motor (SKU: 638242, RobotZone/ServoCity, United States) with a stall torque of 4.12 Nm for our design (2024). The paired motor controller was a SparkFun Motor Driver (14450, SparkFun, United States) (SparkFun, 2024). Without our planetary drive, this motor is able to achieve the desired torque of 2.991 Nm, as per Equation 4, without our planetary gear system, which will only increase the torque output further. Additionally, the motor rotation speed of 26 RPM also allows for a larger range of speeds for the users, since it provides an elbow rotation rate of 0°/s to 52°/s. This rotational speed is based on the speed on the forearm after the planetary gear reduction. This motor also includes an encoder to track position.

#### 2.1.5 Series elastic element

To define our series elastic element, we define two separate actuation angles of motion, θ_*m,*_ and θ_*l*_, the motor angle and load angle, respectively. For a rigid system, these angles will vary due to *N*_*m*_ of the gearbox, as shown in Equation 6.

$$\theta_{l}=N_{m}^{-1}\theta_{m}$$ (6)

**FIGURE 2:**
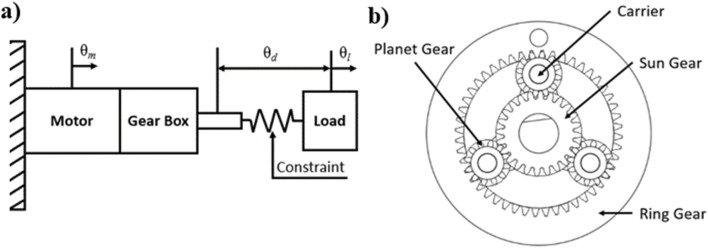
Planetary gear SEA. **(A)** Actuator with spring element between transmission and load (Pratt and Williamson); **(B)** planetary gear schematic.

However, by adding a series elastic element, as proposed by Pratt and Williamson (1995) (Figure 2A), an additional degree of freedom is added to the system (Equation 7). While there is still a single kinematic constraint, often represented by the differential mechanism seen in Equation 7, the spring allows for independent movement between the motor and the load.

$$\theta_{d}=N_{m}^{-1}\theta_{m}-\theta_{l}$$ (7)

Another method for creating a SEA is to use an element that has inherent elasticity, which is also seen in Pratt & Williamson.

A torsional spring was chosen to be integrated into our planetary gear SEA. The spring operates within a range of ±2.109 Nm and is assumed to exhibit good linearity regardless of the direction of rotation. The performance of the SEA is heavily influenced by the characteristics of the spring since the maximum torque is directly linked to the stiffness (Choi et al., 2022). Thus, there is a trade-off between the two. If the spring is too stiff, excessive torque can be generated, leading to user discomfort during use of the exoskeleton. Therefore, the design of the spring must be carefully considered to achieve a balance between maximum torque and precise motor control. For example, stiff springs provide higher torque transmission but low compliance and force control, while softer springs provide higher compliance and force control, but lower torque transmission (Arnaldo Gomes Leal et al., 2016). As such, a variety of springs can be selected based on the application, so for this study we employ a spring that deforms based on the torque requirements of the application (i.e., post stroke assistance) calculated in 2.1.4. The properties of the spring are in Table 4.

**TABLE 4:** Torsional spring specifications.

Specification	Value
Desired max deflection	±20°
Desired max torque	±3 Nm
Spring diameter *D*	29.032 mm
Wire diameter *d*	2.667 mm
Number of turns *N*	7
Spring constant *k*	0.015 Nm/deg

**FIGURE 3:**
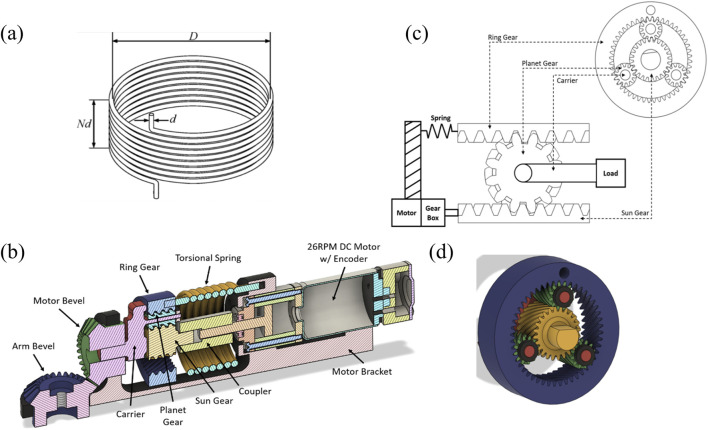
Operating principle. **(A)** Torsional spring; **(B)** Cross-sectional diagram of the proposed torsional spring SEA device; **(C)** Rotary-to-linear motion projection schematic of the planetary SEA system; **(D)** Helical gears used in the planetary gear system.

The mechanical design of the torsional spring used in the SEA can be seen illustrated in Figure 3A. The spring constant, a measure of stiffness, is determined by the spring’s geometry (geometrical diameter D, wire diameter d, and number of coils N) and the material’s elastic modulus as shown in Table 4.

#### 2.1.6 Planetary gear

A planetary gear was introduced to achieve the differential mechanism constraints set by the SEA system. This transmission is composed of a sun gear, planet gears, a ring gear, and a carrier. The planet gears are connected to the carrier which makes the planetary gear an epicyclic gear seen in Figures 2, 3.

The sun gear, through the input shaft, observes the input torque from the source, such as a motor. Planet gears with a carrier connect the sun and the ring gears. The torque output can be determined based on the fixed element in the planetary gear. With the ring gear fixed the transmission ratio ( $i_{r}$ ) is defined by Equation 8:

$$i_{r}=1+\frac{N_{r}}{N_{s}}$$ (8)

where *N*_*r*_ and *N*_*s*_ are the numbers of teeth on the ring gear and sun gear, respectively. From this, the torque can be found using:

$$\tau_{c}+i_{r}\tau_{s}=0$$ (9)

where τ_c_ and τ_s_ are the carrier and sun gear torques, respectively.

The use of a planetary gear allows for compactness, high efficiency, and low backlash. These qualities render planetary gears better suited for SEA-based rehabilitation devices, since the combination allows for a more precise and natural movement control in rehabilitation patients (Card and Lowe, 2024; Park et al., 2023; Moeller et al., 2022; Choi et al., 2022).

Here we select a 3:1 helical planetary gear system. With a 3:1 gear ratio for the planetary gear system, using Equation 9, the max output torque of the system is 12.36 Nm. A 3:1 ratio is achieved by using a gear tooth ratio of 12:24:48 as the planet, sun, and ring gears, respectively. With a maximum output torque of 12.36 Nm, the system can be used for a larger population of users. The bevel gears used in the actuator system were maintained at a 1:1 gear ratio to maintain the output torque seen by the user at 12.36 Nm maximum.

### 2.2 Operating design principle

The planetary gear system, described above, has three parts that rotate relative to the fixed point. The chosen configuration utilizes the sun gear as the input and the carrier as the output to achieve a high gear ratio. The elasticity of the system utilizes a torsional spring between the ring gear and ground (fixed motor bracket). This includes the motor stator being fixed to the motor bracket and the rotor being connected to the sun gear. The DC motor has a speed of 26 RPM and 4.12 Nm of max torque to account for the 2.991 Nm loss torque by the user. The integration of the motor into the system and the component breakdown of the system are shown in Figure 3B.

The torque produced by the motor becomes the input torque for the planetary gear system by connecting the motor rotor to the sun gear, thereby driving the actuator system. The sun gear position is defined by the angular position of the motor rotor and is measured by the encoder. The carrier of the system serves as the output shaft, which connects to the load being moved. This is shown in Figure 3C.

### 2.3 Software control

**FIGURE 4:**
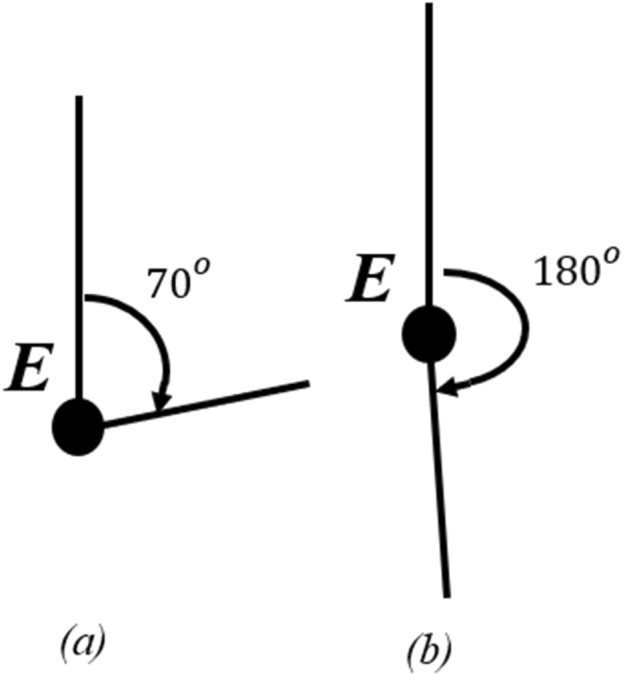
Arm position during rehabilitation: **(A)** start and **(B)** full extension positions.

To control the device a script was written in C++ and uploaded to an Arduino UNO using the PlatformIO IDE. In the script, the code utilizes a proportional-integral-derivative (PID) controller to regulate the motor position. We use the encoder value that is received by the Arduino and then subsequently compute a control signal based on the difference between the current and target positions of the device. The script can include pre-programmed target positions for cyclical rehabilitation and/or testing in order to identify encoder position accuracies relative to the position of the user’s arm. For example, parameters can be input to assume a desired starting position–e.g., 70° angle (Figure 4A) – and end position (e.g., 180° degrees per Figure 4B).

For example, in our code:

u=Kp * e+Kd * dedt+Ki * eint;

where Kp = 8, Kd = 0.05, and Ki = 0.01, and e is the sensory value, along with its derivative, dedt, and integral, eint. Ki can also be set to 0.0 for PD control.

The encoder accuracy was determined to be 0.26° per PID count. This was calculated using the number of pulse cycles per revolution of the motor shaft and the internal motor gear ratio. The PID was tuned by testing the position of the encoder against the desired target position and adjusting the PID components to achieve a critically damped system. This was done to minimize the risk of injury that can occur by rotating past the applicable range of motion and maintaining the movement at a consistent speed. All code is available on our public repository (see Data availability).

### 2.4 Manufacturing and assembly

**FIGURE 5:**
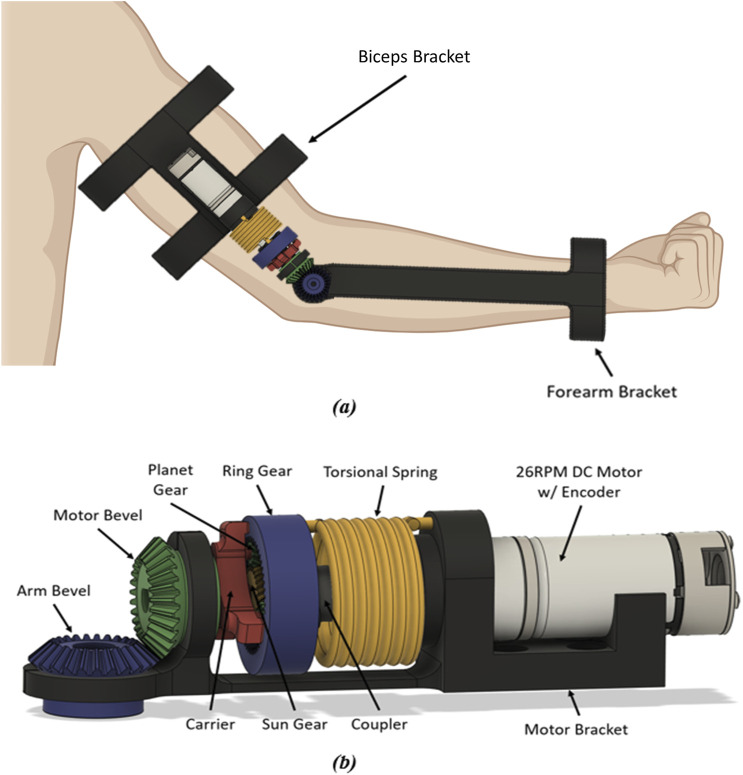
**(A)** Full-model design of the proposed torsional spring SEA device. **(B)** Component view of proposed torsional spring SEA actuator.

Figure 5A shows the fully assembled device based on the above design parameters. Most design components were manufactured using standard PLA plastic, except for the motor and spring elements. PLA is the most common 3D printing material, and is thus selected due to its accessibility and ease of use–however other materials such as ABS, Carbon Fiber reinforced PLA, and other materials can also be used (with some changes in performance) (Rojek et al., 2023; Cornejo et al., 2021). This design also utilized two brackets to connect the actuating device to the user using Velcro straps. Figure 5B shows the non-sectioned view of the components for the proposed SEA actuator system.

An initial passive compliance test was completed to measure the anticipated range of motion under un-powered conditions. This isolates the contributions of the spring mechanism and the planetary gear system. To do so, the bicep bracket was secured, and the forearm bracket was initially positioned at 180°. This position served as the baseline. The forearm bracket was then rotated until the spring became engaged due to the fixed sun gear (motor inactive). The angle between the initial and engaged positions were measured, quantifying the passive system compliance at 34.38°. To isolate the contribution of the planetary gear system, a secondary test was performed by mimicking the initial conditions of the stress test, but stopping the rotation before the spring engaged. This measurement, 15.43°, represented the inherent compliance of the planetary gear system. By subtracting this value from the total compliance measured in the first test, the contribution of the spring mechanism was determined to be 18.95°.

All models were designed in Fusion360 and printed using a Robo R2 and Prusa MK3 3D printers. Design drawings can be found on our GitHub repository.

#### 2.4.1 Bracket design

We include two brackets to attach to the forearm and upper arm. To allow broad usability independent of user morphology, a wide wrist cuff opening is made (Figure 5A). Similarly, the bicep bracket was designed to have a length 95 mm in order to minimize frictional contact with body segments. Both attachment brackets were built with slots to insert Velcro straps to attach to the user. The bracket can be modified in the provided CAD files on our GitHub repository to account for different limb sizes.

#### 2.4.2 Motor bracket

The bracket was designed to prevent slipping of the motor bevel gear which is comprised of a slot specific to the motor size (Figure 5B). The arm bevel gear location was built with a gap to allow for assembly of the gears. This gap also allows for some tolerance that allows the gears to be placed in the brace. A redesign of the motor bracket eliminated the gap built into the motor bevel location, as it could cause unwanted deformation.

#### 2.4.3 Planetary gear

To eliminate slippage of the teeth when a load is applied, a helical gear system is included (Figure 3D). In this way, the teeth gradually engage, resulting in smoother meshing compared with spur gears. The helical gear teeth meshing also allows for a higher load capacity without tooth wear, due to the large effective contact area. This is especially advantages for 3D printed systems, since the plastic is more likely to wear than metallic systems. Additionally, planet stops were also added as shown in black in Figure 3D, to stop the thrust of the planet gears.

#### 2.4.4 Printing

We manufactured all components using PLA. However, each component of the system incurs different loading (e.g., gears vs. bracket), and so various characteristics can be used. Specifically, we used various infill settings when manufacturing (Table 5). Effectively, the infill is the percentage of volume used by plastic inside the part–the remaining volume is air. Similarly, the number of perimeters that was used to create the components was three for all parts.

**TABLE 5:** Component infil percentages.

Components	Infill (%)
Bicep, Forearm, Motor Brackets	30
Sun Gear, Ring Gear, Motor Bevel, Arm Bevel	60
Coupler, Carrier, Planet Gear, Planet Stops	100

### 2.5 Performance tests

**FIGURE 6:**
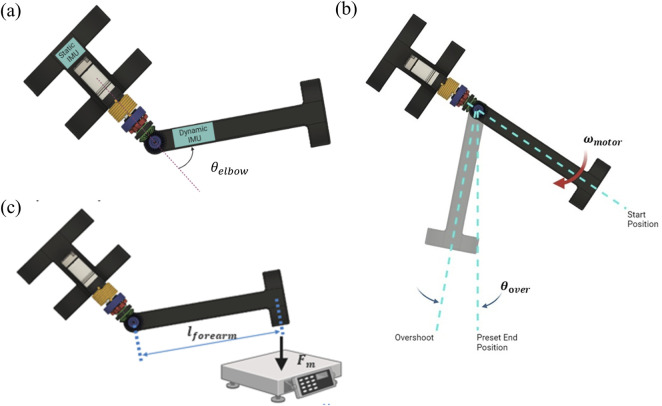
Joint compliance test setups. **(A)** IMU placement on exoskeleton, **(B)** dynamic compliance test, **(C)** Joint angle vs torque output at the elbow test.

To quantify the compliance of the SEA actuator system using the angle of the elbow joint ( $\theta_{elbow}$ in Figure 6A), three tests were designed. The goal was to evaluate how the SEA joint responded to static and dynamic loads, as well as how it responded to different torques present at the elbow. Static loading is an essential component of these assistive devices as it often used to determine system behavior under maximal loading scenarios (Ji et al., 2020; Kong et al., 2024). In our case, we will understand the compliant effect on the SEA/Exoskeleton joint under the effects of the motor. Similarly, dynamic testing provides ground-truth for measuring changes of the device during use (Xiang et al., 2023; Zheng et al., 2021) – which can affect safety, risks, range of motion, etc. Our dynamic tests will assess the mechanical systems under varying speeds and torques, to determine how compliance is distributed.

All three tests used a similar setup. Two inertial measurement units (IMUs) were placed on the exoskeleton to collect data to calculate the elbow joint angle during each test–this could be compared to the encoder position. Specifically, the angular velocity data recorded by the gyroscope built into the IMU was of interest, as it could be used to determine the angle of the elbow. One IMU was placed on the bicep bracket, which remained fixed during all three tests. The fixed IMU acted as a ‘fixed’ reference for other IMU, which was placed on the forearm bracket which would be free to rotate (Figure 6A).

#### 2.5.1 Static compliance test

The purpose of the static compliance test was to examine how the elastic elements in the joint responded to static loads, similar to the passive compliance test. To accomplish this test, the forearm bracket was manually pushed until the spring engaged to measure the angle of elbow compliance contribution of the gear only ( $\theta_{gear}$ ). The elbow angle was calculated within MATLAB through an integration of the raw angular velocity data recorded by the IMU. Four trials following this procedure were run to obtain an average gear compliance. Following this, a similar process was followed but instead of stopping at the instance of spring engagement, the forearm bracket was pushed past the instant of spring engagement until the spring started to linearly deform. We define this as the total system compliance ( $\theta_{total}$ ). From there, the spring compliance ( $\theta_{spring}$ ) can be calculated by subtraction (Equation 10).

$$\theta_{spring}=\theta_{total}-\theta_{gear}$$ (10)

#### 2.5.2 Dynamic compliance test

For this test, the rotational speed of the motor was varied by adjusting the amount of voltage provided to it by a DC power supply. An Arduino code was programmed to have the motor rotate the forearm bracket approximately 60° from its starting position at full extension, then shut off the motor. It was hypothesized that as the forearm rotated to this preset angle, it would overshoot due to the momentum, compliance and elasticity of the gear and spring (Figure 6B). Furthermore, with increasing forearm speeds, it was expected that there would be greater overshoots before the forearm settled at its steady state.

The angular velocity of the forearm was recorded with the IMUs, then integrated within MATLAB to calculate the relative elbow angle. Percent overshoot (i.e., the comparison between the overshoot angle and final steady state angle), and settling time (i.e., the duration between motor shut down and when the forearm stopped at its steady state angle) were calculated within MATLAB as well. In this test, steady state was defined as the final angular position of the forearm after the it coming to a complete rest. Since two parameters in the percent overshoot and settling time were known, second order system Equations 11 and 12 could be used to calculate the damping ratio and natural frequency of the elastic joint under different speeds. Specifically,

$$\zeta =\frac{-\ln\;\left(\frac{\%os}{100}\right)}{\sqrt{\pi^{2}+{\left(\ln\left(\frac{\%os}{100}\right)\right)}^{2}}}$$ (11)

$$\omega_{n}=\frac{4}{T_{s}\zeta }$$ (12)

where %OS is the percent overshoot, ζ is the damping co-efficient, Ts is the settling time, and $\omega_{n}$ is the natural frequency of a second order dynamic response.

Five sets of data were collected, each corresponding to a different motor supply voltage: 12, 14, 16, 18 or 20 V. Each set consisted of five trials at that specific supply voltage. In addition to the calculations described above, the raw angular velocity data and integrated elbow angle data were graphed within MATLAB.

The MATLAB code used to plot the data also extracted the maximum angular velocity of the forearm, angular velocity at the peak overshoot angle, and the final steady state angle. Lastly, the code calculated the overshoot angle, amplitude of the subsequent oscillation, percent overshoot, and settling time. These characteristics, along with the damping ratio and natural frequency calculated with Equations 11 and 12, were recorded for each individual trial.

#### 2.5.3 Joint angle v. torque output at the elbow test

For the final test, we measured how varying amounts of elbow torque affects the elbow joint angle when met with resistance. The motor controlling the elbow was coded to rotate the forearm from a set starting position into a force scale. The scale was positioned between the coded start and stop positions, preventing the forearm from reaching its target. Consequently, the force reading on the scale is equal to the amount exerted by the forearm (Figure 6C).

Equation 13 utilizes the formula for torque along with the length of the forearm ( $l_{forearm}$ ) and the force reading ( F ) to calculate the exact torque output of the elbow joint.

$$\tau_{elbow}=l_{forearm}\cdot F$$ (13)

Getting the forearm to exert increasing forces, and therefore increasing torque outputs, was accomplished by increasing the DC voltage supplied to the motor from 10 to 18V, in increments of two. Additionally, the IMUs were also collecting data for analysis within MATLAB. Using these data, the change in angle from the moment of contact with the scale to the instant the motor stopped was calculated. We hypothesized that if the torque output at the elbow was increased, so would the change in elbow angle due to the compliant joint. We collected this data using motor voltages of 10V, 12V, 14V, 16V, and 18V.

#### 2.5.4 Finite element analysis (FEA) of gearing

Three finite element analysis (FEA) tests were performed in Fusion 360 for the relevant components of the SEA. When performing FEA on 3D printed components the results are dependent on the percent infill of the plastic, the infill structure and the material properties of the specific plastic used. These tests shown here are performed with the mechanical properties of PLA (Travieso-Rodriguez et al., 2019).

Three FEA analyses are performed in this study in order to support our designs. Case 1 was performed on the bevel gear contact between the SEA and the forearm bracket. This test consisted of fixing the SEA bevel gear and applying 3 Nm of torque to the forearm. This torque was used based on the required 2.991Nm torque, as per Equation 4 – see Section 2.1.4. The next two cases were performed on the planetary gear system of the SEA. Case 2 applied the same 3 Nm torque on the sun gear, while the planetary gears were pinned restricting tangential movement but allowing rotational and the ring gear was fixed. The last case, Case 3, the torque was applied to the arm tied to the planetary gears with the planets and sun pinned in the same fashion and the ring gear fixed.

## 3 Results

### 3.1 Static compliance

**FIGURE 7:**
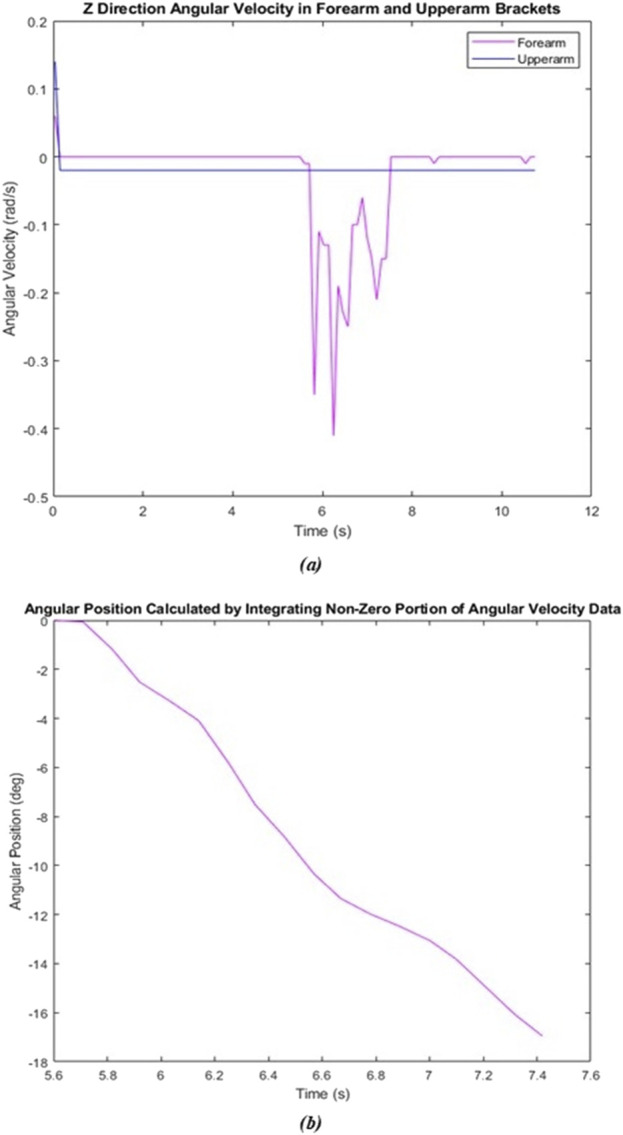
Static test–gear compliance trial 1. **(A)** Raw angular velocity data. **(B)** Angular position data calculated through MATLAB.

As described above, a total of eight trials were conducted during this test, four to calculate an average planetary gear compliance and four to calculate an average total system compliance. MATLAB was utilized to integrate the angular velocity data from the IMUs to obtain angular position data. Both sets of all four trials were plotted against time to obtain a visual of the forearm’s motion over time. Figure 7 shows one such pair of graphs.

The average compliance contribution of the planetary gear and the average total system compliance were calculated to be 14.80° and 22.22° respectively. Utilizing Equation 10, the average spring compliance under a static load was 7.42°.

### 3.2 Dynamic compliance

**FIGURE 8:**
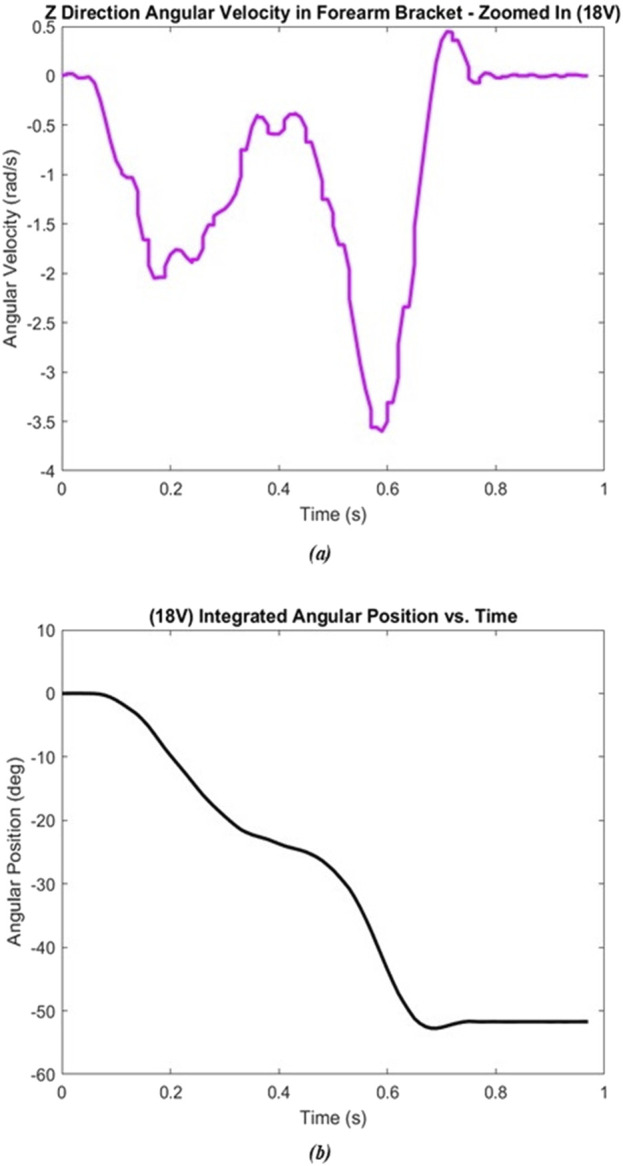
Dynamic test – 18V trial 1. **(A)** Raw angular velocity data. **(B)** Angular position calculated through MATLAB.

**FIGURE 9:**
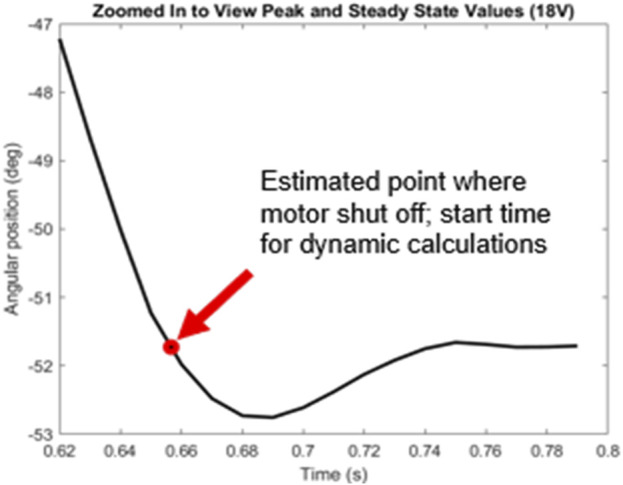
Zoomed in angular position data for 18V–trial 1.

Similar to the static compliance test, the angular velocity and integrated angular position data were both plotted against time (Figure 8). At all voltage levels, the forearm overshot the preset angle as expected, oscillated once, and then settled to a final position. Thus, our second order assumption for the system was observed. The red circle in Figure 8A shows where the system recovers from the overshoot. Because the motor rotated the forearm clockwise (i.e., negative direction), the positive angular velocity indicates that the forearm rotated in the opposite direction due to compliant elastic effects. The same is seen in Figure 8B when observing with the angular position data. A closeup of this dynamic even is shown in Figure 9.

Here, the overshoot caused by the compliance and elasticity of the SEA actuator is readily seen, with a peak angle of approximately −52.7° occurring at about 0.685 s. This point is used for the subsequent dynamic analysis results. This point thus defines where the motion was dependent on the forearm’s rotation and independent from the motor (since it was stopped there). Thus, t = 0 for all data below, was defined as the instant the motor shuts off as shown in Figure 9.

The full data table can be found in our Supplementary Material. Table 6 contains the truncated averaged values for each voltage level. For the input voltages 12–18 we can see an increasing angular velocity, overshoot angle, oscillation amplitude, %OS, and other related factors that we expect to see in a second-order dynamic response. One variation however is when we applied a 20 V input to the motor. The 20 V input is above the specifications for the motor which is likely causing these changes in dynamic performance.

**TABLE 6:** Dynamic response vs input voltage.

Input voltage (V)	Max forearm speed (rad/s)	Overshoot (deg)	Oscillation amplitude (deg)	% overshoot	Settling time (s)	Damping ratio	Natural frequency (rad/s)
12	−1.8460	−0.0911	−0.0940	0.2059	0.0585	0.8991	91.5598
14	−2.3600	−0.3501	−0.3764	0.7993	0.1584	0.8458	33.4168
16	−3.0220	−0.7173	−0.7357	1.4187	0.1427	0.8048	36.7994
18	−3.4180	−0.9626	−1.0055	1.9321	0.1256	0.7827	41.2393
20	−3.4075	−0.2664	−0.2808	0.5304	0.0586	0.8643	109.6748

### 3.3 Joint angle v. torque output at the elbow

**FIGURE 10:**
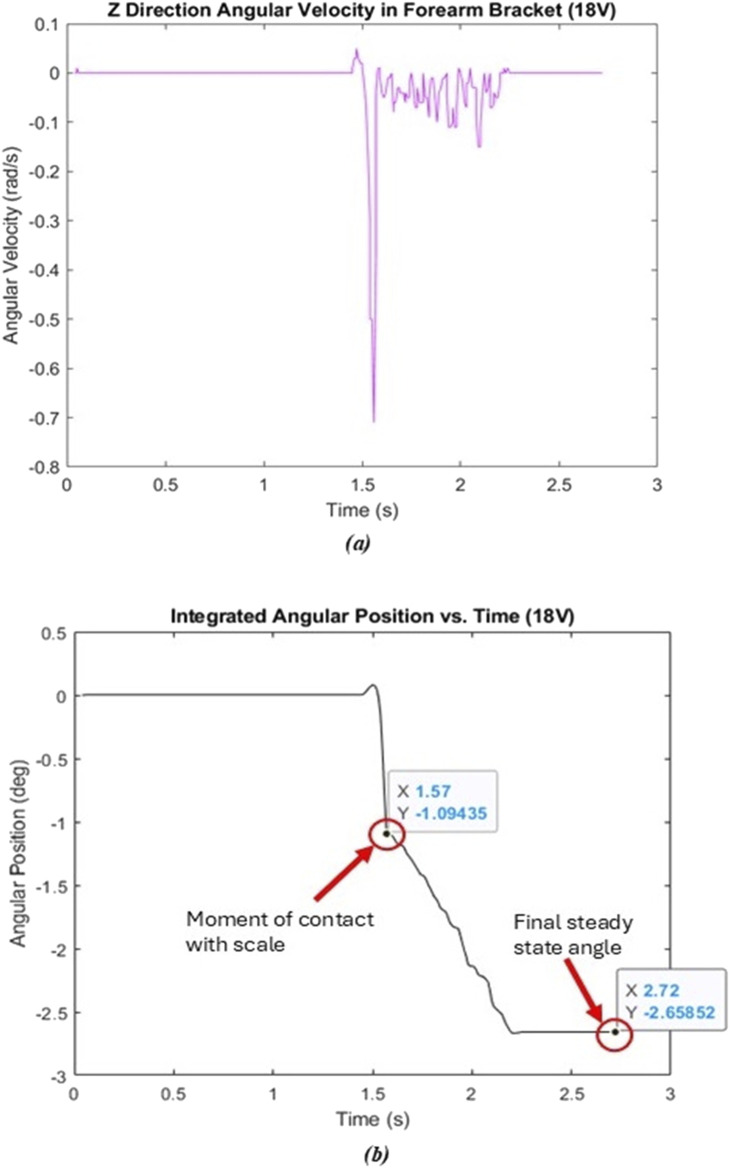
Joint angle v. torque test – 18V. **(A)** Raw angular velocity data. **(B)** Angular position calculated through MATLAB.

Figure 10 shows a sample plot where 18V was used as the input voltage to the motor. The general shape of both plots was seen in each of the five trials at different voltage levels. To evaluate the change in angle ( Δθ ) associated with the differing torques, the joint angle at the moment of contact with the scale was subtracted from the steady state angle once the motor stopped. These two points are circled in red in Figures 10A, B. For this trial, Δθ was calculated to be −1.5642° (e.g., Δθ = −2.65852° - (−1.09435°)). The Δθ for each voltage level is summarized in Table 7.

**TABLE 7:** Torque and angle values for each input voltage.

Voltage (V)	Force (g)	Force (lb)	Forearm length (ft)	Torque (ft lb)	Δθ (deg)
10	59	0.1301	0.67615	0.0879	−1.6501
12	28	0.0617	0.67615	0.0417	−0.9397
14	30	0.0661	0.67615	0.0447	−0.9482
16	34	0.0750	0.67615	0.0507	−0.9798
18	50	0.1102	0.67615	0.0745	−1.5642

The force measured by the forearm pushing on the force scale and the torque calculated based off that is also detailed in Table 7. Generally, the data in the table show an increasing trend from 12 to 18V, where an increased voltage–and thus motor torque–corresponds to an increasing force recorded by the sensor, and an increasing Δθ due to compliance. The trial with a 10V input however, does not match this trend. Evidently, an increasing torque and force are produced, which also creates a large Δθ . This is due to the voltage/torque relationship of the motor.

### 3.4 FEA analysis

For FEA Case 1, the normal stress was captured for the X, Y, and Z-axis, where the max stress for the bevel gears was 60.2 MPa, 57.9 MPa, and 50 MPa, respectively. These stresses all occurred at high stress contact points between the two gear teeth. The max displacement resulting from the stress was 0.14 mm. Case 2 and 3 analyzed the normal stresses in the X and Y-axis with the Z-axis forces being minimal reaction forces. For the second case the max stress in the X and Y direction were 82.9 MPa, and 81.7 MPa. Displacement mainly occurred in the sun gear coupler at 0.129mm, but within the gears at 0.036 mm. Case 3 was similar, but consisted of the most stress occurring in the arm transferring torque into the planet gears with the highest stresses being 36.1 MPa, and 58.3 MPa. This had the max displacement of 0.118 mm in the arm and 0.005 mm in the gears. Images for each axis are available in the Supplementary Material.

## 4 Discussion

Our work demonstrates an openly available 3D printed series elastic actuated planetary gear joint for an elbow exoskeleton. This device was designed to provide a low-cost compliant system to support broadly accessible applications in rehabilitation-based robotics. We estimate the total cost of the device to be $75.69.

We designed the system such that the planetary geared motor could achieve an output torque of 12.36 Nm, whereas our calculated loss torque was 2.99Nm (i.e., required torque to assist a post-stroke patient). This includes an encoder accuracy of 0.26° per PID count. The motor torque was transmitted through a 3:1 helical planetary gear.

### 4.1 Static testing

In a preliminary design-based qualitative test, a total compliance of 34.38° and a spring compliance of 18.95° were estimated. Quantitative testing showed that the true total compliance was 22.22° respectively while the average spring compliance was 7.42°. Thus, 74.9% of the compliance was measured through the planetary gear system, while 25.1% of the compliance was due to the spring. Notably, the compliance of the joint was distributed between the planetary gear design and the spring. For example, using only a spring in this case would not provide as much compliance in the design, since the planetary gear system accounted for relatively much more flexibility in the system. This allows the exoskeleton to mimic the compliance in a human joint, to enable a soft interface, and allow the natural compliance in the joint to subsist (Gallay et al., 1993).

In human biology, the compliance of the elbow joint changes with age and diseased states. For example, although muscle co-activations can remain similar throughout aging, the elasticity in joint control degrades of time (Valour and Pousson, 2003). By changing the parameters of our 3D printed system, we can then scale the compliance of the exoskeleton to match the characteristics of the individual.

### 4.2 Dynamic compliant performance

There are several important trends that can be observed from Table 6. The data in the first two columns demonstrates that increasing the voltage supplied to the motor resulted in a corresponding increase in the angular velocity of the forearm. As predicted, the overshoot increased as the speed of the motor increased. Consequently, the amplitude of the oscillation and percent overshoot also increased. In terms of percent overshoot, this means that with increased speed, the overshoot was a larger percentage of the forearm’s steady state angle. Finally, the average damping ratios decreased as the forearm speed increased. This was to be expected since damping ratios closer to 1 indicate a more stable system. If the overshoot was larger, the system would be less stable and therefore have a lower damping ratio. The deviant data occurred when the DC motor was pushed to 20V. Since the motor was only rated for 12V, it is likely that the motor experienced secondary effects, such as high levels of back electro-motive force, and affected its second order behavior.

By examining the sensitivity of the motor’s rotational speed to the voltage change, we can see that from 12 to 14 V, 14–16 V, 16–18 V, and 18–20 V the speed changes by 0.257, 0.331, 0.198, and −0.005 radians per second per volt. In other words, the 16 V motor input produced the highest change in torque/speed, while a 20 V input reduced the speed relative to 18 V input. In any case, the torque, speed, and overshoot increased with voltage up to 18 V, which corresponds with our hypotheses that greater movement speeds would increase the compliant effect in the joint. As such, it is essential that the correct motor is selected for these exoskeletons, as compliant performance can change based on loading. In this study, we designed our system to operate in a range similar to the maximum assistance torque needed for a paretic post-stroke limb. In clinical applications where inter-subject functional ability is highly varied, or intra-subject ability changes with time, the torque characteristics of the assisting motor can be scaled on the individual’s ability or progress in recovery.

### 4.3 Joint angle vs. torque

Our results support the hypothesis that increasing the torque output at the elbow would also increase the change in elbow angle after the forearm due to compliance. As expected, the change in elbow joint angle increased as the torque increased. In other words, as the system experienced a higher torque, the excess loading was transmitted through the compliant interface, causing the measuring bending (i.e., change in angle) motion. However, the data collected when 10 V was being supplied to the motor did not follow this trend. In fact, the torque and angle change measured were greater than any other data point. Likely, the lower rotational rate created a larger torque, since electric motors produce higher stall torques.

Thus, this test demonstrated that the compliance in the joint reacts to the torque and speed of the motor. In other words, higher loading in the joint is transferred through the compliant interface (torsion spring in this case). This is an important characteristic, as it therefore limits loading of the robotic system onto the human joint, or an overly rigid interaction across the human-machine interface. Future tests will explore how this loading affects the human joint compliance. Notably, the characteristics of the torsional spring can be designed to emulate the compliant characteristics of the biological joint.

There are other limitations on our design of course, if too great of a torque is applied to the system, additional compliance can manifest through mechanical deformation, which is not the intended use of the system. In future applications with human-based tests, the configuration of the mechanical design must consider the potential for mechanical deformity, as this work here only considers the assistance of the exoskeleton on human movement, rather than the effect of human movement on a static exoskeleton.

### 4.4 FEA performance

The performance of the PLA plastic showed varied stress and displacements during FEA for the gear teeth and surfaces. For the purpose of this analysis only the gear teeth and surface layers were observed because of the complexities of the internal structures of the gears when not at 100% infill. Using the estimated torque from Section 2.1.4 (i.e. 2.991 Nm), the simulated resulting stresses within the gear systems are below the flexural strength of 83 MPa of PLA. Thus, PLA is able to withstand a 3 Nm torque based on our “assistive” applications and are relevant for this design. To allow for cases where more torque is needed, different 3D printing materials with greater flexural strength–such as carbon fiber reinforced nylon with a flexural strength of 175 MPa–could be used. Material selection in this way allows for a greater level of assistance with the SEA and in different types of rehabilitation modalities.

## 5 Conclusion

This study designed and developed a 3D-printed planetary gear SEA joint exoskeleton for elbow rehabilitation. The proposed design addresses the limitations of inherent stiffness in traditional actuator systems by incorporating a torsional spring into an openly available mechanism to support rehabilitation research.

In future studies, it will be pertinent to further explore how segment rigidity supports the joint compliance effect, specifically in human-based experimental applications. For example, although this presented SEA joint design demonstrate compliance, there are limitations in knowing its effect in human-assisted rehabilitation. This is especially true in cases where there is movement resistance (emulating static compliance outcomes in the SEA), or where the human overpowers the motor effect (either through resistance or spasticity). As a result, our future studies will explore these relationships to demonstrate how these SEA exoskeleton designs can facilitate movement in healthy humans, followed by clinical applications in post-stroke participants.

Similarly, we have so far only evaluated the SEA design using commonly used PLA plastics. We aim to explore how these dynamic effects change with material type. For example, uniformity in the design (i.e., PLA used for all segments, gears, etc.) may not be an ideal framework for the exoskeleton. By having a heterogeneous material design, we can promote more compliance in certain parts of the system (i.e., joint), while limiting the compliance in other parts (i.e., segments). In this way, optimization of the joint compliance can be controller based on the application and user ability, so that a cohesive frame work that supports limb posture, while providing dynamic compliance, is achieved.

In other future studies, we will explore how variable stiffness and personalization of the stiffness in can affect performance outcomes (e.g., fatigue, range of motion, etc.). Additionally, we will instrument the exoskeleton with additional sensors enhance user feedback as a part of use to provide more precise position and torque control. Our ultimately long-term goal will investigate the use of these 3D printed SEA exoskeletons in post-stroke volitional control rehabilitation, and performance optimization in high-risk jobs, such as warehouse workers.

In this study we have shown that compliant 3D printed SEA-based joints can be developed to support low-cost human-robot interaction applications such as in assistive rehabilitation exoskeletons.

## Data Availability

The datasets presented in this study can be found in online repositories. The names of the repository/repositories and accession number(s) can be found below: https://github.com/COMANDLab/SEA_Exoskeleton.
