# Supplementary material for: OpenSEA: a 3D printed planetary gear series elastic actuator for a compliant elbow joint exoskeleton
Source: Front Robot AI. 2025 Feb 28;12:1528266. doi: 10.3389/frobt.2025.1528266 (PMC11906680; doi:10.3389/frobt.2025.1528266)

Supplementary Data

Static Compliance Test Data Table

Trial #	Gear Compliance Angle (deg)	Total Compliance Angle (deg)		
1	-16.9538	-22.1391		
2	-14.2695	-23.2077		
3	-14.725	-20.0507	**Spring Compliance**	7.4234
4	-13.2411	-23.4855		
Avg	-14.7974	-22.2208		

Dynamic Compliance Test: complete Data Table

Voltage (V)	Max Forearm Speed (rad/s)	Omega at Peak Angle (rad/s)	Overshoot (deg)	Oscillation Amplitude (deg)	Final Angle (deg)	% Overshoot	Settling Time (s)	Damping Ratio	Natural Frequency (rad/s)
12	-1.94	-0.04	-0.1518	-0.1662	-49.6067	0.3061	0.0871	0.8789	52.2509
12	-1.79	0.02	-0.1948	-0.1948	-42.5794	0.4575	0.0895	0.8638	51.7371
12	-1.84	-0.04	-0.0745	-0.0745	-40.3448	0.1846	0.0465	0.8948	96.1397
12	-1.75	0.02	-0.0086	-0.0086	-40.3477	0.0213	0.0278	0.9374	153.4981
12	-1.91	0.03	-0.0258	-0.0258	-42.8716	0.0601	0.0417	0.9208	104.1734
14	-2.20	0.06	-0.4927	-0.5414	-42.8802	1.1491	0.1723	0.8179	28.3835
14	-2.24	-0.16	-0.5500	-0.6016	-43.9315	1.2520	0.1992	0.8126	24.7107
14	-2.02	-0.07	-0.4985	-0.5128	-43.1724	1.1546	0.2244	0.8176	21.8013
14	-2.53	-0.01	-0.1404	-0.1576	-49.6411	0.2828	0.0916	0.8816	49.5323
14	-2.81	0.06	-0.0688	-0.0688	-43.5677	0.1578	0.1043	0.8991	42.6561
16	-2.97	-0.03	-0.6274	-0.6589	-47.8849	1.3102	0.2113	0.8097	23.3789
16	-3.02	0.13	-0.7133	-0.7305	-51.5891	1.3827	0.1148	0.8062	43.2182
16	-3.05	-0.12	-0.9053	-0.9167	-51.4860	1.7583	0.1334	0.7895	37.9811
16	-3.04	0.00	-0.6274	-0.6532	-50.8500	1.2338	0.1089	0.8135	45.1498
16	-3.03	0.12	-0.7133	-0.7191	-50.6409	1.4086	0.1450	0.8050	34.2691
18	-3.60	0.14	-1.0285	-1.0972	-51.7266	1.9883	0.1134	0.7802	45.2129
18	-3.17	-0.30	-1.1488	-1.1803	-48.9306	2.3478	0.1536	0.7667	33.9662
18	-3.21	-0.01	-0.8107	-0.8222	-50.2398	1.6137	0.1093	0.7957	45.9949
18	-3.64	-0.15	-0.9397	-0.9769	-48.7788	1.9264	0.1284	0.7826	39.8062
18	-3.47	-0.43	-0.8852	-0.9511	-49.6067	1.7845	0.1231	0.7884	41.2160
20	-3.51	-0.24	-0.5185	-0.5185	-49.4835	1.0479	0.0745	0.8234	65.2075
20	-3.52	0.13	-0.2979	-0.3552	-51.1164	0.5829	0.1019	0.8535	45.9937
20	-3.28	-0.27	-0.1977	-0.1977	-50.8099	0.3890	0.0360	0.8702	127.6807
20	-3.32	0.08	-0.0516	-0.0516	-50.7755	0.1016	0.0220	0.9099	199.8172

DYNAMIC COMPLIANCE TEST: TRUNCATED TABLE OF AVERAGE VALUES

Voltage (V)	Max Forearm Speed (rad/s)	Absolute Value Omega at Peak Angle (rad/s)	Overshoot (deg)	Oscillation Amplitude (deg)	Final Angle (deg)	% Overshoot	Settling Time (s)	Damping Ratio	Natural Frequency (rad/s)
12	-1.8460	0.030	-0.0911	-0.0940	-43.1500	0.2059	0.0585	0.8991	91.5598
14	-2.3600	0.034	-0.3501	-0.3764	-44.6386	0.7993	0.1584	0.8458	33.4168
16	-3.0220	0.062	-0.7173	-0.7357	-50.4902	1.4187	0.1427	0.8048	36.7994
18	-3.4180	0.068	-0.9626	-1.0055	-49.8565	1.9321	0.1256	0.7827	41.2393
20	-3.4075	0.066	-0.2664	-0.2808	-50.5463	0.5304	0.0586	0.8643	109.6748

JOINT ANGLE V. TORQUE AT THE ELBOW TEST DATA TABLE

Voltage (V)	Force (g)	Force (lb)	Forearm Length (ft)	Torque (ft lb)	Angle at impact (deg)	Final angle (deg)	Δθ (deg)
10	59	0.1301	0.67615	0.0879	-1.2748	-2.9250	-1.6501
12	28	0.0617	0.67615	0.0417	-2.5812	-3.5208	-0.9397
14	30	0.0661	0.67615	0.0447	-1.3264	-2.2746	-0.9482
16	34	0.0750	0.67615	0.0507	-1.6444	-2.6242	-0.9798
18	50	0.1102	0.67615	0.0745	-1.0944	-2.6585	-1.5642

FINITE ELEMENT ANALYSIS

Bevel Gear Displacement

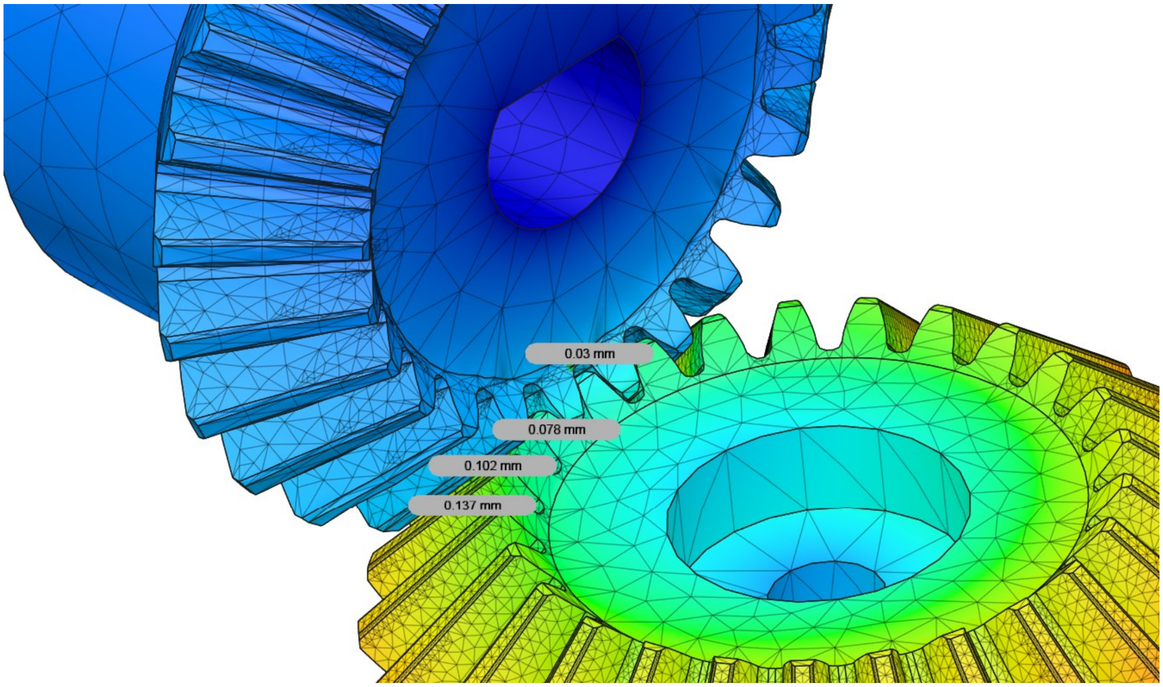

FEA: Bevel Gear X-Axis Stress

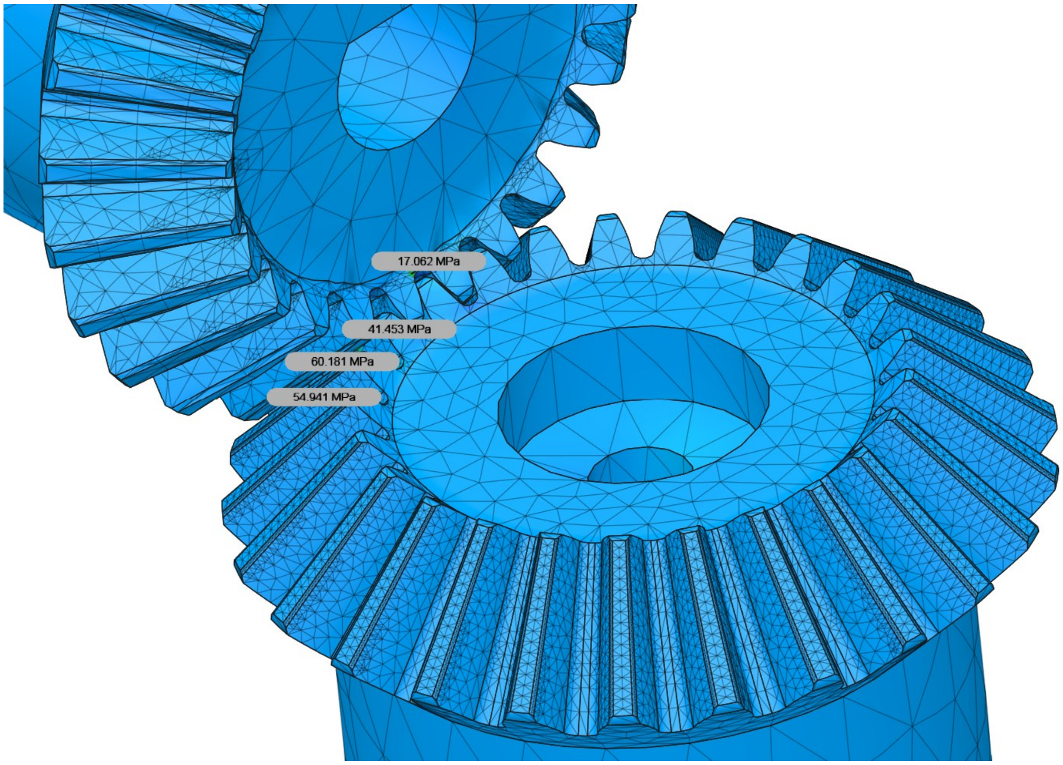

FEA: Bevel Gear Y-Axis Stress

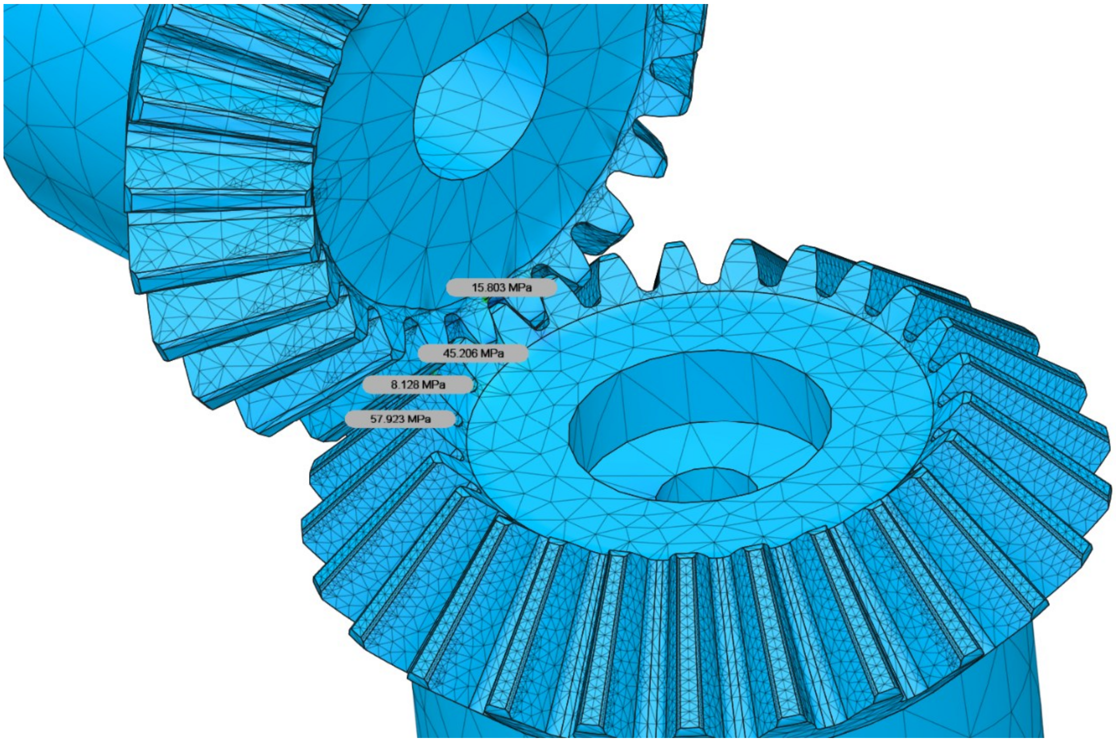

FEA: Bevel Gear Z-Axis Stress

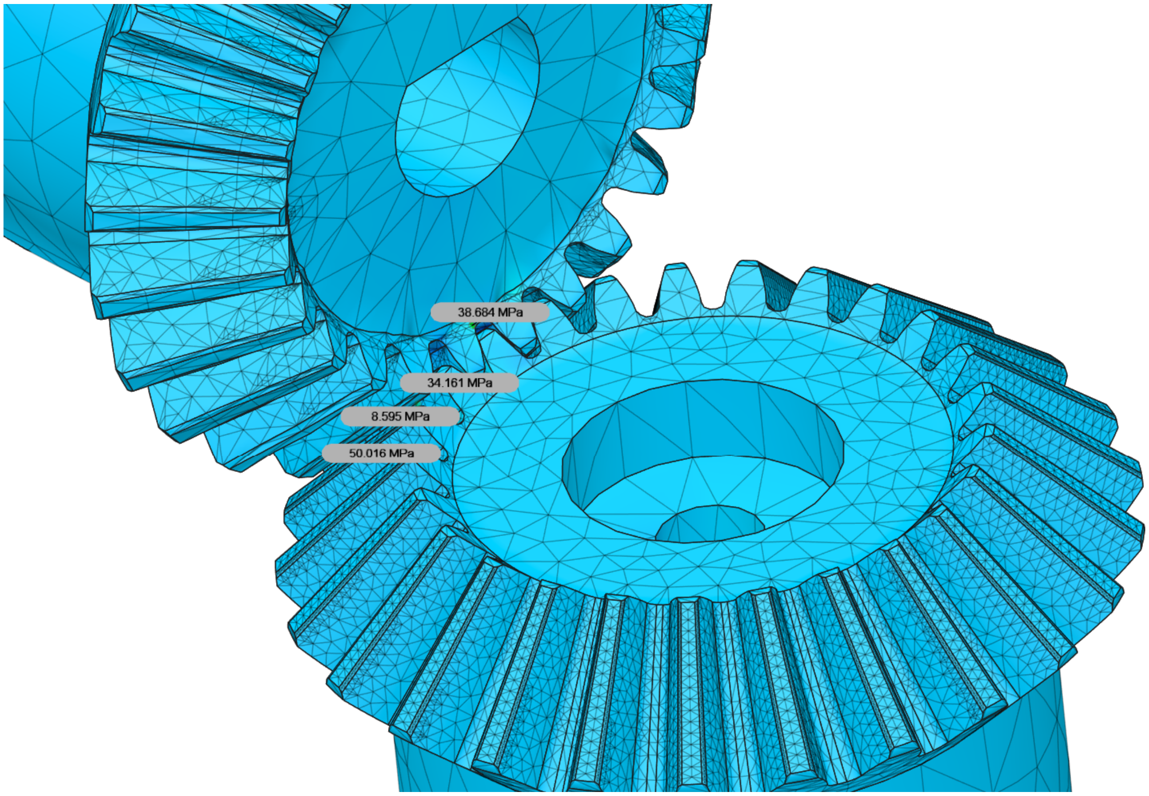

FEA: SUN GEAR TORQUE DISPLACEMENT

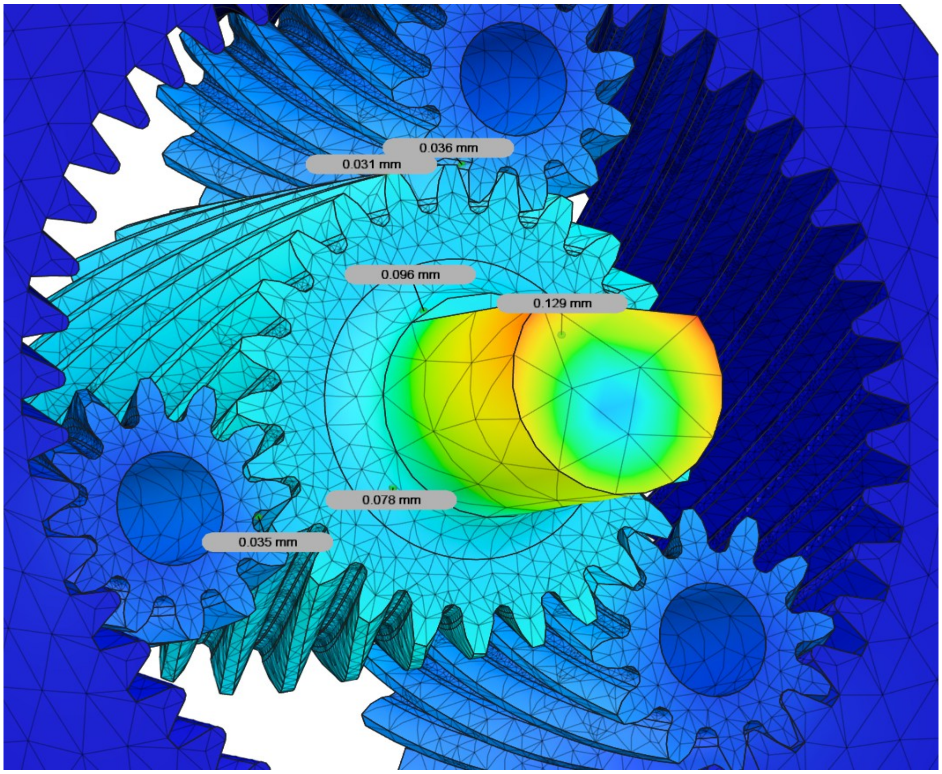

SEA: SUN GEAR X-AXIS STRSS

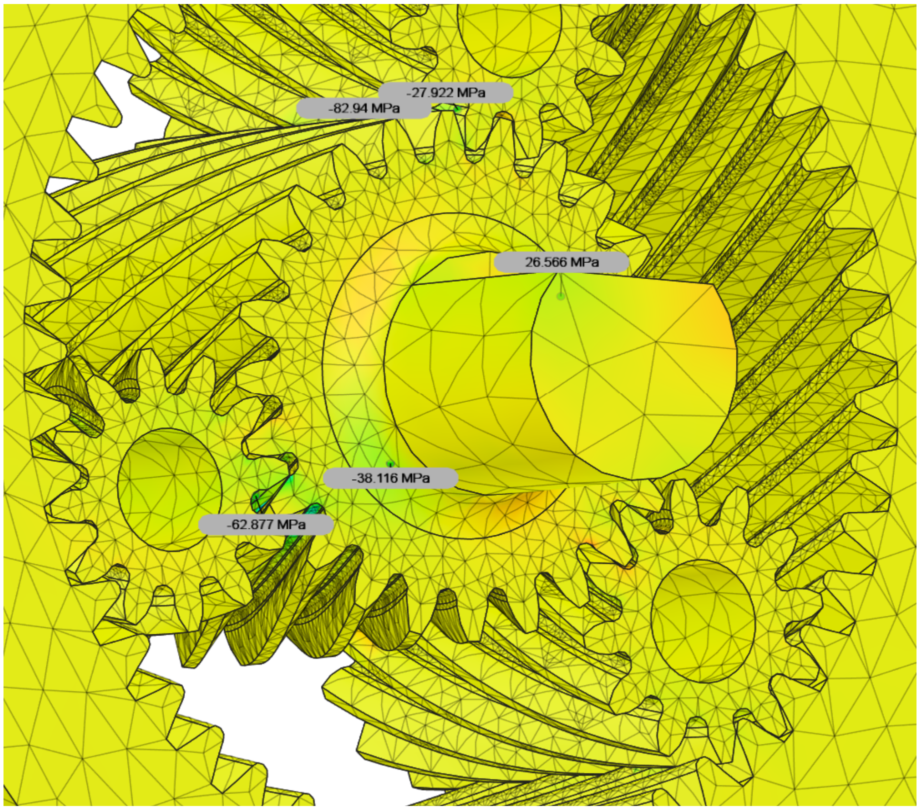

FEA: SUN GEAR Y-AXIS STRESS

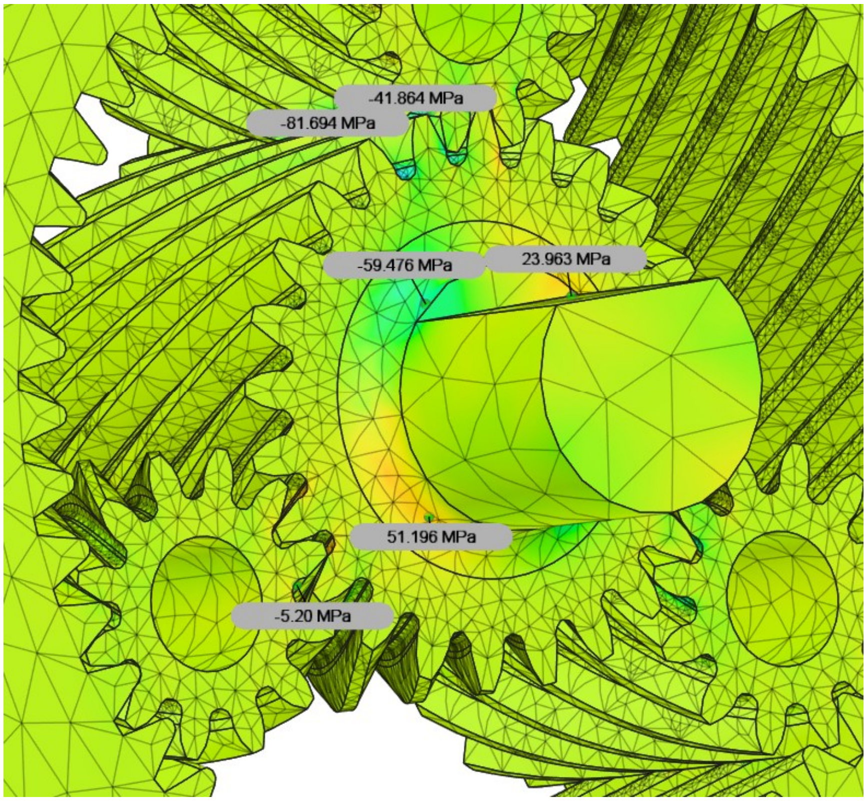

FEA: ARM TORQUE DISPLACEMENT

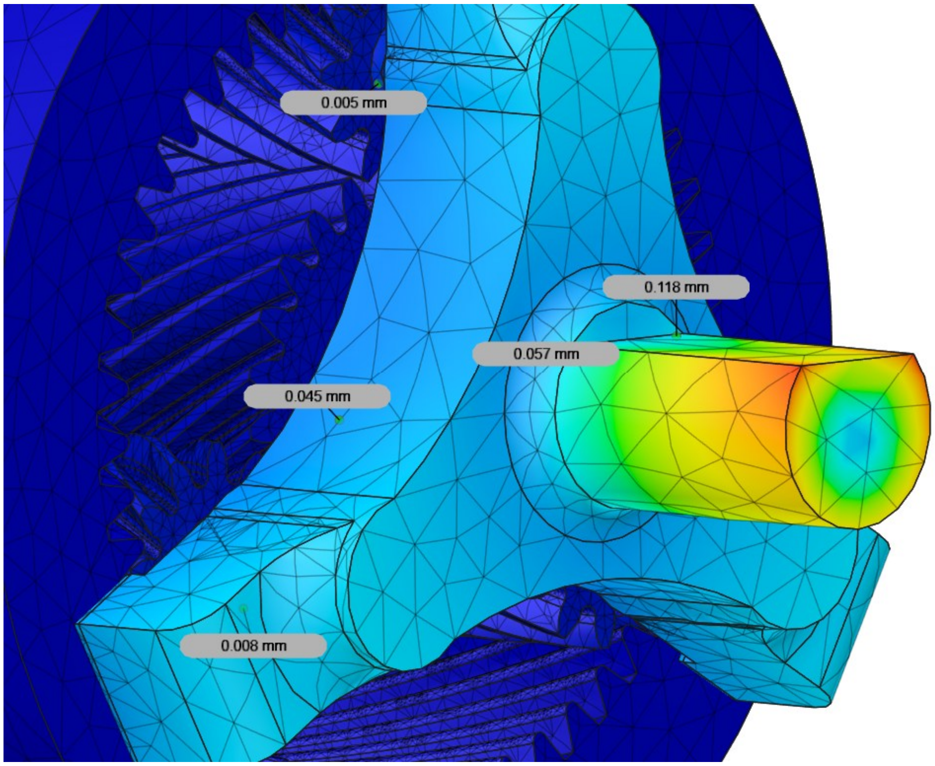

FEA: Arm X-Axis Stress

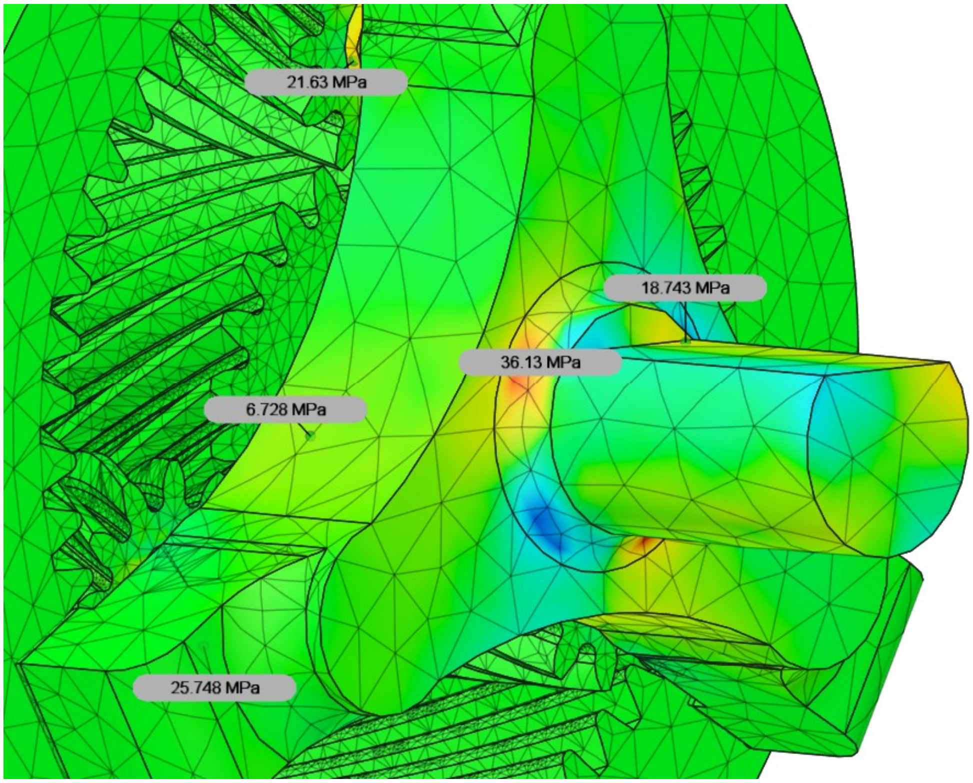

FEA: Arm Y-Axis Stress

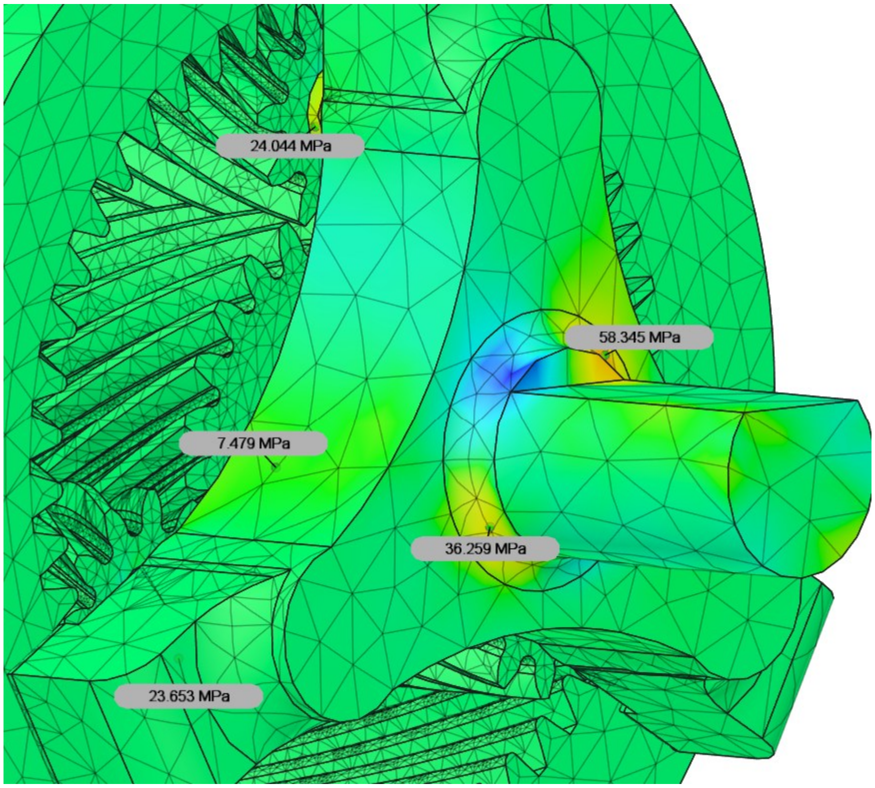

Supplement: Supplementary file 1 [file DataSheet1.pdf]
